# An ecoacoustic dataset collected on the island of Cyprus in the Mediterranean Basin biodiversity hotspot

**DOI:** 10.1038/s41597-025-04807-1

**Published:** 2025-03-19

**Authors:** Christos Mammides, Christina Ieronymidou, Harris Papadopoulos

**Affiliations:** 1 Nature Conservation Unit, Frederick University, Nicosia, 1036 Cyprus; 2 Southeast Asia Biodiversity Research Institute, Chinese Academy of Sciences & Center for Integrative Conservation, Xishuangbanna Tropical Botanical Garden, Chinese Academy of Sciences, Mengla, Yunnan 666303 China; 3 BirdLife Cyprus, Nicosia, 2340 Cyprus; 4 Department of Electrical Engineering, Computer, Engineering and Informatics, Frederick University, Nicosia, 1036 Cyprus

**Keywords:** Biodiversity, Agroecology

## Abstract

There is growing interest in using novel technologies for large-scale biodiversity monitoring. Passive acoustic monitoring (PAM) represents a promising approach for surveying vocalizing animals. However, further development of PAM methods is needed to improve their accuracy. The availability of extensive ecoacoustic datasets from biodiverse areas can facilitate this development. In this study, we present a large ecoacoustic dataset (1.58 TB) collected at sixty-one study sites on the island of Cyprus between March and May 2023. The dataset comprises >313,000 audio files, representing over 5,200 hours of recordings. It can be used for a range of applications, such as developing and refining species identification algorithms, acoustic indices, and protocols for processing acoustic data to exclude non-focal sounds, e.g., those produced by human activities. It can also be used to explore fundamental ecological questions. To facilitate its use, the complete dataset has been made available on the Hugging Face repository and the ARBIMON platform, operated by Rainforest Connection^TM^, which offers a range of free tools for ecoacoustic analyses.

## Background & Summary

Human activities are accelerating biodiversity loss^1^. National and international efforts are being made to address this global challenge. In December 2022, countries worldwide committed to the ambitious targets of the Kunming-Montreal Global Biodiversity Framework (GBF), aiming at halting and reversing biodiversity loss by 2030^2^. A crucial component of the framework is the proper monitoring of progress, including the monitoring of biodiversity patterns^3^. To achieve this, it is essential to develop effective monitoring technologies capable of assessing biodiversity across large spatial and temporal scales^3^.

When considering vocalizing animals, such as birds, passive acoustic monitoring (PAM) methods have been proposed as a promising approach^4^. PAM methods involve collecting acoustic data using autonomous recording units, which can then be analyzed to extract ecologically meaningful information about the biodiversity in a specific area of interest^5^. Multiple techniques for analyzing acoustic data exist, primarily distinguished by whether the analysis aims to identify the species present or summarize the area’s acoustic environment (soundscape)^6^.

Species identification techniques usually involve the supervised training of a machine learning algorithm to recognize the acoustic patterns of specific species using a set of annotated acoustic samples. This approach is gaining popularity as algorithms become more accurate. Nowadays, off-the-shelf species identification algorithms are available^7,8^, reducing implementation barriers by requiring fewer programming skills. Furthermore, specialized platforms such as ARBIMON (https://arbimon.org/) allow users to train species identification algorithms through user-friendly interfaces needing no programming skills. However, species identification techniques have been shown to perform sub-optimally on certain occasions^9^, e.g., when monitoring entire animal communities with multiple overlapping vocalizing species or communities with numerous background noises, making focal signal detection difficult.

An alternative approach for analyzing ecoacoustic datasets has been using acoustic indices^10^, which are essentially mathematical formulae that summarize the acoustic environment of an area of interest^11^. The rationale behind this approach is that more biodiverse areas will have more complex and heterogeneous acoustic environments. Hence, by quantifying acoustic heterogeneity, we can make inferences about the biodiversity in an area of interest^12^. Over sixty such indices are currently available^11^, with several shown to correlate with species diversity^10^ and diversity of biological sounds^11^. However, recent research has highlighted that the indices’ performance is, in many cases, inconsistent^13,14^ and varies considerably across regions^14,15^ depending on the specific characteristics of the soundscapes in those areas^6,16^. For instance, it was shown that several of the indices tend to be less accurate in more biodiverse regions^14^, e.g., tropical environments, likely due to the higher number of species. Also, other studies have shown that non-focal sounds, such as those produced by human activities or non-biological processes (e.g., wind and rain), considerably affect the indices’ performance^16,17^. When working with small ecoacoustic datasets, it’s possible to manually process the data and remove or filter non-focal acoustic signals. However, this is not practical when dealing with large ecoacoustic datasets, which is often the case when monitoring biodiversity over large spatial and temporal scales. Recently, automated algorithms have been proposed for preprocessing audio files and cleaning up ecoacoustic datasets, such as datasets affected by rain^18^. However, this is still a field under development requiring more robust and comprehensive solutions.

**Fig. 1:**
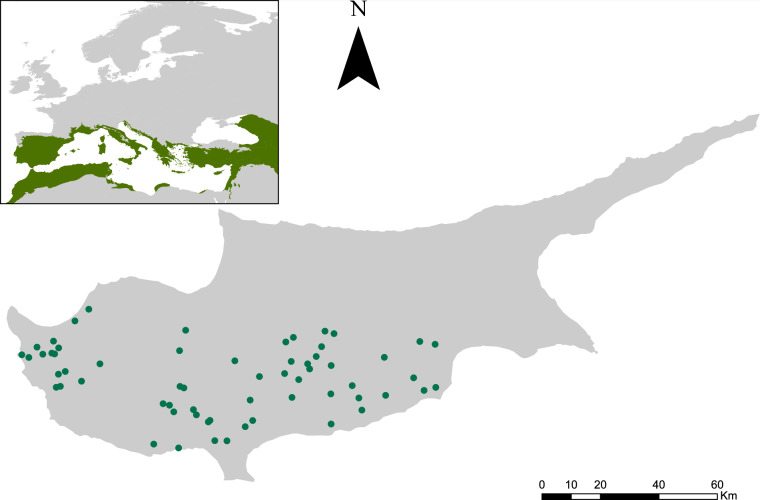
Map of Cyprus indicating the locations of the sixty-one study sites at which the acoustic data were collected between March and May 2023. The inset map illustrates in green colour the Mediterranean Basin biodiversity hotspot.

The above challenges compromise the effectiveness of passive acoustic monitoring methods, underscoring the need for further development to support wider adoption and use by conservationists and other stakeholders. Accelerating such development could be facilitated by the availability of suitable datasets from biodiverse areas. In this article, we present a large ecoacoustic dataset (1.58 TB) collected at sixty-one sites on the island of Cyprus (Fig. 1), part of the Mediterranean Basin biodiversity hotspot^19^. The dataset, collected between March and May 2023, comprises 313,197 audio files, corresponding to over 5,200 recordings. It can be used for a range of applications, such as (a) developing or improving species recognition algorithms, (b) further testing and refining acoustic indices, and (c) developing new tools for removing signals produced by non-biological processes or by non-focal species. Additionally, as indicated by its inclusion in the Worldwide Soundscape project (https://ecosound-web.de/ecosound_web/collection/index/106), the dataset can be used to answer fundamental ecological questions^20^, e.g., by analyzing the acoustic patterns over time and space and relating them to other variables of interest widely available, such as land cover data^21^. The dataset was collected as part of the BIOMON project funded by the European Commission (https://cordis.europa.eu/project/id/101090273), aiming at exploring the effectiveness of acoustic indices in monitoring bird communities in biodiverse sites^6,22^.

## Methods

Cyprus is the third-largest island in the Mediterranean Sea known for its rich biodiversity, including avian diversity^23^. More than 400 bird species have been recorded on the island thus far, with about two-thirds of them being migratory species. Several bird monitoring programs are in place on the island (https://birdlifecyprus.org/monitoring-birds/), with experienced volunteer bird surveyors conducting ground surveys at regular intervals. One of these programs is the Common Birds Monitoring Scheme (CBMS), coordinated by BirdLife Cyprus, contributing to a larger pan-European scheme (https://pecbms.info/). For the purposes of the CBMS monitoring program, bird surveys are conducted annually between March and June at ~100 study sites.

**Fig. 2:**
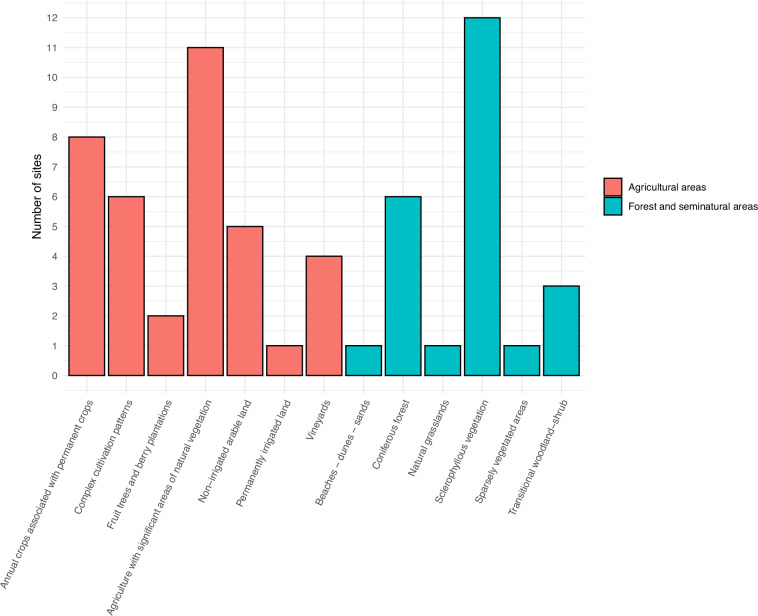
A histogram illustrating the major land cover class at each site based on the most recent CORINE Land Cover map (2018).

We collected acoustic data at sixty-one of those sites (Fig. 1) in low-intensity agricultural areas and forest and seminatural areas (Fig. 2) between March 1^st^ and May 26^th^, 2023. To identify the land cover at each site, we used ArcGIS Pro (version 2.9) to overlay the GPS locations of each site with the most recent Corine Land Cover map (2018) available at https://land.copernicus.eu/en/products/corine-land-cover. We then used fifteen Song Meter Mini acoustic recorders (Wildlife Acoustics) purchased for the purposes of BIOMON to record audio files at the sixty-one sites. The fifteen recorders were deployed sequentially across the sites. At each site, a recorder was installed on a tree at a height of ~1.5 meters for approximately one week (mean = 7.2 days, min = 7 days, max = 9 days). The device was programmed using the Song Meter mobile app to record 30 audio files per hour, each one minute long^24^, for 24 hours per day, resulting in an average of 5,134 audio files per site (min = 4,344 files, max = 6,684 files, standard deviation = 399 files). A 30/60 duty cycle was selected in order to keep the size of the dataset manageable while at the same time ensuring that rarer sounds were still captured. Following best practice guidelines for long-term ecoacoustic monitoring^24^, the following recording settings were used: (a) sample rate = 48000 Hz, (b) recording mode = highest quality, and (c) channel gain = 18 dB. All audio files were saved in the recommended^24^ WAV format, which is also the default format for the Song Meter Mini recorder.

## Data Records

All acoustic files have been made freely available on the Hugging Face repository^25^ at 10.57967/hf/2613. The complete dataset consists of 61 zipped folders, each corresponding to a different site (Fig. 1). The folders were named using the following convention: “FolderNumber. SiteID – RecorderID”. For example, folder “38. T063 Pedoulas – SMA11420” represents the 38^th^ folder out of the 61 available and contains all the audio files (n = 5,098) recorded at the site “T063 Pedoulas” using the Song Meter Mini recorder with the ID “SMA11420”. An Excel file, named “Ecoacoustic Dataset_Cyprus_Study Sites.xlsx,” has also been made available on the dataset’s page on the repository^25^, outlining for each zipped folder: (a) the folder number, (b) the location at which the audio files were recorded (i.e., site name), (c) the ID of the Song Meter Mini recorder used, (d) the date the recorder was deployed and retrieved, along with the total duration in days, (e) the number of audio files recorded at each site, (f) the geographic coordinates (latitude & longitude) of the exact location the recorder was placed at each site, (g) the elevation, and (h) the corresponding land cover class^21^ (Level 1 & 2) at each site (Fig. 2) according to the most recent Corine Land Cover map (2018) available at https://land.copernicus.eu/en/products/corine-land-cover. A more detailed description of each land cover class can be found here: https://land.copernicus.eu/content/corine-land-cover-nomenclature-guidelines/html

Additionally, each zipped folder on Hugging Face contains a metadata summary text file associated with the recording session at each site (as generated by default by the Song Meter Mini recorder). Specifically, the summary file includes for each audio file the following information: (1) the date and time the audio file was recorded, (2) the latitude & longitude, (3) the power (i.e., the measured voltage of the recorder’s batteries), (4) the temperature (in degrees Celsius), and (5) the number of full-spectrum “.wav” files recorded during the preceding minute. Further details regarding the default metadata recorded by Song Meter Mini can also be found at: https://www.wildlifeacoustics.com/uploads/user-guides/html/Mini2-HTML5/en/sd-card-contents.html.

Once recorded, audio files are automatically named by each Song Meter Mini and saved on the device’s SD memory card. Wildlife Acoustics uses the following naming convention: “RecorderID_YYYYMMDDD_HHMMSS.wav,” specifying the recorder’s ID and the exact date and time the audio file was created. For example, the audio file named “SMA11420_20230502_060000.wav” was recorded using the Song Meter Mini recorder with the ID SMA11420 on May 2^nd^, 2023, at 06:00 AM. We have chosen to retain the original file names as several existing tools can read this commonly used format and automatically extract the information about the date and the time each recording was made. In total, we recorded 313,197 audio files (1.58 TB), representing over 5,200 hours of recordings. The audio files can be accessed for each site separately by downloading the corresponding zipped folder from the Hugging Face repository.

## Technical Validation

To assess the quality of the audio files, we first checked all folders for audio recordings that were either too short or lacked acoustic information owing to a machine malfunction. Based on the recording settings we describe in the methods section, each one-minute-long audio file should be between 5.6 and 5.8 MB. Therefore, files shorter than this size were inspected and removed when faulty. We also manually removed any files recorded during the installation and retrieval of the devices in the field to exclude sounds related to equipment setup, such as human voices and trampling vegetation. As an additional validation step, we used the *multiple_sounds* function (with default settings) of the“soundecology”^26^ package in the R Programming Language^27^ to calculate for each audio file six acoustic indices commonly used in the literature for capturing acoustic patterns related to biodiversity^10,14,28^. Those indices are: (1) the Acoustic Complexity Index (ACI), (2) the Acoustic Diversity Index (ADI), (3) the Acoustic Evenness Index (AE), (4) the Bioacoustic index, (5) the Acoustic Entropy Index (H), and (6) the Normalized Difference Doundscape Index (NDSI). A detailed description of each index and how it is calculated can be found in the recent Acoustic Index User’s Guide^29^ developed by Bradfer‐Lawrence and colleagues: https://ecohack.shinyapps.io/Acoustic_Index_Users_Guide/. When calculating the six indices, if an audio file resulted in an NA value, the file was inspected and removed from the dataset in case of malfunction. The output of the six acoustic indices for each audio file can be found in the Excel file (“Ecoacoustic Dataset_Cyprus_Acoustic Indices R.xlsx”; 32.8MB) on the dataset’s page of the repository^25^. The first column in the Excel file corresponds to the site ID, the second to the name of the audio file (in the format ‘RecorderID_YYYYMMDD_HHMMSS.wav’), followed by the Recorded ID, recording date, time, and the six acoustic indices.

## Usage Notes

To further facilitate the reuse of the ecoacoustic dataset presented in this study, we have also made it available on the ARBIMON platform (https://arbimon.org/p/biomon)^30^. ARBIMON is operated by Rainforest Connection^TM^ and is designed to host and analyze acoustic data for ecological applications. The platform provides a range of freely available tools for analyzing ecoacoustic files^19^ and allows the inspection of individual files, which can be downloaded separately or in batches.

## Data Availability

No code was used to produce the acoustic data. The six acoustic indices were calculated using the default settings of the *multiple_sounds* function in “soundecology”^26^ package in the R Programming Language^27^.
